# High-Throughput Screening and Proteomic Characterization of Compounds Targeting Myeloid-Derived Suppressor Cells

**DOI:** 10.1016/j.mcpro.2023.100632

**Published:** 2023-08-14

**Authors:** Johannes Krumm, Elissaveta Petrova, Severin Lechner, Julia Mergner, Hans-Henning Boehm, Alessandro Prestipino, Dominik Steinbrunn, Marshall L. Deline, Lisa Koetzner, Christina Schindler, Laura Helming, Tobias Fromme, Martin Klingenspor, Hannes Hahne, Jan-Carsten Pieck, Bernhard Kuster

**Affiliations:** 1 Chair of Proteomics and Bioanalytics, Technical University of Munich, Freising, Germany; 2 Global Research & Development, Discovery and Development Technologies, Discovery Pharmacology, Healthcare Business of Merck KGaA, Darmstadt, Germany; 3 Bavarian Center for Biomolecular Mass Spectrometry at Klinikum rechts der Isar (BayBioMS@MRI), Technical University of Munich, Munich, Germany; 4 Global Research & Development, TIP-Oncology & Immunooncology, Myeloid Cell Research, Healthcare Business of Merck KGaA, Darmstadt, Germany; 5 OmicScouts GmbH, Freising, Germany; 6 Chair of Molecular Nutritional Medicine, TUM School of Life Sciences, Technical University of Munich, Freising, Germany; 7 Global Research & Development, Discovery and Development Technologies, Global Medicinal Chemistry, Healthcare Business of Merck KGaA, Darmstadt, Germany; 8 Global Research & Development, Discovery Technologies, Computational Chemistry & Biologics, Healthcare Business of Merck KGaA, Darmstadt, Germany; 9 Else Kröner Fresenius Center for Nutritional Medicine, Technical University of Munich, Freising, Germany; 10 ZIEL Institute for Food & Health, Technical University of Munich, Freising, Germany; 11 Bavarian Biomolecular Mass Spectrometry Center (BayBioMS), Technical University of Munich, Freising, Germany

**Keywords:** proteomics, post-translational modifications, chemical proteomics, myeloid-derived suppressor cells, high-throughput screening, reactive oxygen species

## Abstract

Myeloid-derived suppressor cells (MDSC) are a heterogeneous cell population of incompletely differentiated immune cells. They are known to suppress T cell activity and are implicated in multiple chronic diseases, which make them an attractive cell population for drug discovery. Here, we characterized the baseline proteomes and phospho-proteomes of mouse MDSC differentiated from a progenitor cell line to a depth of 7000 proteins and phosphorylation sites. We also validated the cellular system for drug discovery by recapitulating and identifying known and novel molecular responses to the well-studied MDSC drugs entinostat and mocetinostat. We established a high-throughput drug screening platform using a MDSC/T cell coculture system and assessed the effects of ∼21,000 small molecule compounds on T cell proliferation and IFN-γ secretion to identify novel MDSC modulator. The most promising candidates were validated in a human MDSC system, and subsequent proteomic experiments showed significant upregulation of several proteins associated with the reduction of reactive oxygen species (ROS). Proteome-wide solvent-induced protein stability assays identified Acyp1 and Cd74 as potential targets, and the ROS-reducing drug phenotype was validated by measuring ROS levels in cells in response to compound, suggesting a potential mode of action. We anticipate that the data and chemical tools developed in this study will be valuable for further research on MDSC and related drug discovery.

## Highlights

• (Phospho-)proteome profiling of differentiated myeloid-derived suppressor cells.
• Phenotypic screen of 21,000 small molecules to identify novel MDSC modulators.
• Reduction of reactive oxygen species identified as potential mode of action.
• Chemical proteomics reveals Acyp1 and Cd74 as potential target proteins.

## Graphical Abstract

**Figure:**
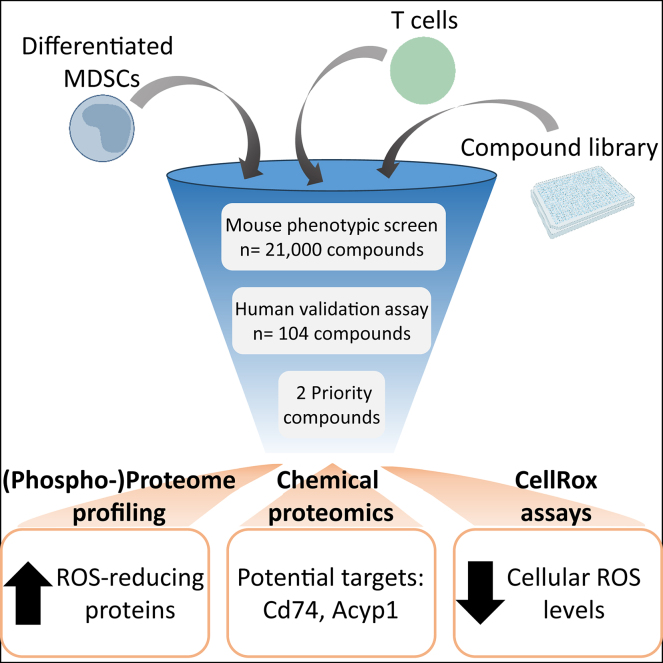

## In Brief

Myeloid-derived suppressor cells (MDSCs) counteract the immune system when fighting cancer. Krumm *et al*. screened 21,000 compounds in T-cell coculture system in search for modulators of MDSC. They identified Acyp1 and Cd74 as potential targets of the most interesting compound by chemical proteomics. Characterization of the cellular response by (phospho-)proteomics and functional assays revealed upregulation of cellular reactive oxygen species metabolism as a likely mode of action of this MDSC modulator.

Treatment with immune checkpoint inhibitors, targeting cytotoxic T lymphocyte–associated protein 4 (CTLA4), programmed cell death protein-1 (PD-1) or its ligand (PD-L1) has provided substantial benefits to some patients with different tumor entities. This indicates that successful clinical responses can be obtained by blocking inhibitory mechanisms which suppress T cell functions in the tumor niche (1, 2, 3). However, only few patients show a sustained respond to immune checkpoint inhibitor, pointing out that other immunosuppressive factors might contribute to tumoral immune escape. The identification and modulation of such factors could open up new opportunities for innovative cancer immunotherapeutic strategies.

The cellular immune landscape of the tumor microenvironment not only includes T cells but also myeloid cells, which are either tissue-resident or recruited from the circulation and which further shape antitumor immunity (4). Such cells are often over-represented across several cancer types, and while some of them exert anti-tumor activity, by sustaining T cell activation and promoting tumor cell phagocytosis, others impair T cell–mediated killing and facilitate tumor growth (5). In particular, myeloid-derived suppressor cells (MDSC) have been recognized as key players in inhibiting T cell functions in several cancer types (6, 7, 8). MDSC are considered to be immature myeloid cells, derived from dysregulated myelopoiesis (9), and pathologically activated in response to stimuli produced within the tumor microenvironment (10). MDSC are normally absent under physiological conditions but can be identified in cancer patients and mouse cancer models (11). The frequency of such cells usually increases along the disease stage (12) and is correlated with poor prognosis (13). In addition, MDSC have been associated with mechanisms of resistance towards targeted therapies (14) and immunotherapies (15, 16, 17). Phenotypically, MDSC are highly heterogeneous and broadly classified into monocytic-MDSC and granulocytic-MDSC (or polymorphonuclear-MDSC, PMN-MDSC), according to the expression of certain surface markers that are preferentially expressed in the respective myeloid lineages (8). While a comprehensive analysis of specific MDSC markers is complex, and topic of an increasing number of studies (6, 18), MDSC remain best identified by their ability to block T cell functions, which is usually evaluated *via* assessing T cell proliferation and cytokine production upon coculture (19). Known mechanisms that MDSC operate to achieve such inhibitory effects include (but are not limited to) the production of reactive oxygen/nitrogen species (ROS/RNS) (20, 21), activity of arginase-1, and inducible nitric oxide synthase (iNOS) (22) as well as modulation of purinergic signaling (23), expression of PD-L1 (24), and secretion of immunosuppressive cytokines such as interleukin-10 (IL-10) and tumor growth factor-β (25).

Given their pro-tumorigenic, immunosuppressive properties, MDSC are an attractive target cell population in immuno-oncology, and efforts are currently made to identify new strategies to alleviate MDSC-dependent immunosuppression (26). Beyond a direct interference with the mechanisms cited above, few targets have been identified that are relevant for MDSC biology but can be of interest when developing therapeutic interventions. For example, signal transducer-activator of transcription-3 has been shown to induce expression of PD-L1 (27) as well as vascular endothelial growth factor, thereby facilitating MDSC accumulation in the tumor microenvironment (28). The modulation of these and other pathways, which are highly interconnected, is currently under investigation, but despite promising preclinical data, only marginal benefit has been observed so far in the clinical setting. Consequently, new studies to identify innovative and underexplored MDSC-relevant targets are awaited. Intriguingly, it has also been shown that the effects of certain targeted therapies already in clinical use, might, at least in part, rely on their activity on immune cells, including MDSC (29). Epigenetic drugs, for example histone deacetylase (HDAC) inhibitors, are known to exert multiple effects on tumor cells (30), but they can also abrogate the MDSC inhibitory phenotype (31, 32). Deeper mechanistic studies to understand the effects of HDAC inhibitors on MDSC at molecular level are still missing.

A major obstacle that limits the identification of new MDSC targets and pathways (and the activity of small molecules that can modulate them) is the availability of sufficient amounts of cells, as well as the possibility to maintain them in culture. Recently, a model has been described, in which murine hematopoietic progenitors expressing a NUP98/HOXB4 transgene, could be successfully differentiated towards MDSC-like immunosuppressive cells in the presence of granulocyte-macrophage colony stimulating factor (GM-CSF) and IL-6 (33). Such immortalized progenitor cells (referred to as “NUP”) can be readily kept in culture, providing a convenient source of biomass, suitable for high-throughput applications and mechanistic studies. In this work, we used (phospho)-proteomics to characterize NUP-derived MDSC (referred to as NUP-MDSC) cells at the proteomic level. In addition, we describe a high-throughput screening strategy for the identification of novel chemical entities that can restore the ability of T cells (in the presence of NUP-MDSC) to proliferate and secrete interferon-γ (IFN-γ). This may pave the way for the development of new drug candidates that enhance antitumor immunity. We performed such a screen involving about 21,000 compounds to identify hits. Proteomic and functional characterization of one particularly interesting compound identified two candidate targets and pointed to modulation of ROS activity in cells as a potential mode of action.

## Experimental Procedures

### Mouse NUP-MDSC Differentiation Protocol

Mouse NUP cells were maintained in cultivation medium containing RPMI 1640 medium supplemented with 2 mM glutamine, 1 mM sodium-pyruvate, 10% FCS premium, 1 mM nonessential amino acids, 50 μM ß-mercaptoethanol, 50 units/ml penicillin/streptomycin, 20 ng/ml mIl6 (BioLegend #575708, and 100 ng/ml mouse granulocyte/macrophage-colony stimulating factor (mSCF, BioLegend #576304). Cells were passaged every 2 to 3 days and seeded with 200,000 cells/ml. To initiate MDSC differentiation, 1 × 10^5^ cells/ml were seeded in MDSC differentiation medium containing the same supplements as the cultivation medium, except that mSCF was replaced with 20 ng/ml mGM-CSF. Cells were incubated for 4 days without medium change at 37 °C and 5% CO2. Cells in suspension as well as loosely attached cells were harvested, and the expression of MDSC surface markers was checked by FACS.

### Flow Cytometry for Surface Markers

The cells of interest were taken up at the final concentration of 1 x 10^6^ cells/ml in PBS (Life Technologies #14190) plus 2% Fetal bovine serum (FBS, PAN Biotech #P30-1502) and seeded (200 μl/well) into a 96-well plate (Thermo Fisher Scientific #249570). The plate was centrifuged 300*g* for 5 min at 4 °C, the cells were washed twice, and then incubated for 30 min at 4 °C with the respective antibodies. For NUP cells and NUP-MDSC: PerCP anti-mouse/human CD11b (clone M1/70, BioLegend #101229), FITC anti-mouse Ly6C (clone HK1.4, BioLegend #128006), APC anti-mouse CD11c (clone N418, BioLegend #117310), and PE anti-mouse Gr-1 (clone RB6-8C5, BioLegend #108407). For PMN-MDSC: PE anti-mouse/human CD11b (clone M1/70, BioLegend #101208) and APC anti-human CD15 (Biolegend #323008). For human CD34+ cells, FITC anti-human CD34 (BD #555821) was used. The cells were centrifuged and washed again twice in PBS plus FBS, and then data was acquired using iQue Screener Plus (IntelliCyt). Data analysis was done using FlowJo software (https://www.flowjo.com/).

### Arginase Assay

Arginase catalyzes the conversion of arginine to urea and ornithine. The urea produced specifically reacts with the assay substrate to generate a colored product, proportional to the arginase activity. Cells were washed with PBS and lysed for 10 min in 10 mM Tris–HCl, pH7.4 containing protease inhibitors and 0.4% (w/v) Triton X-100. Lysates were centrifuged at 13,000*g* for 10 min, and supernatant was used for analysis. Arginase activity was analyzed using the Arginase Activity Assay Kit (Sigma, MAK112) according to manufacturer’s protocol. Lysates were untreated or treated with titrated amounts of the arginase inhibitor (3R,4S)-3-amino-1-[(2S)-2-aminopropanoyl]-4-(3-boronopropyl)pyrrolidine-3-carboxylic acid. Absorbance (430 nm) was analyzed in an Infinite M1000 Pro (Tecan) plate reader after 120 min of incubation. Arginase activity was determined as follows: arginase activity = absorbance-blank/(urea standard-H_2_O Control)∗(1 mM∗reaction volume ∗10³/(incubation time in min∗sample volume (μL))). One unit of arginase is the amount of enzyme that will convert 1.0 μmole of L-arginine to ornithine and urea per minute at pH 9.5 and 37 °C.

### iNOS Assay

NUP cells or NUP-MDSC (100,000 cells/well in AIM-V medium) were treated with or without titrated amounts of the iNOS inhibitor piperidin-2-ylideneamine for 2 hours. Intracellular NO was stained with DAF-2 DA kit (Enzo, ALX-620-056-M001) according to manufacturer’s protocol, and mean fluorescence intensity was analyzed in a Guava easyCyte 8HT (Millipore) FACS machine.

### T cell Proliferation Assay

384-well plates (Greiner #781090) were coated with anti-mouse CD3 (Clone 145-2C11, BioLegend #100302) and anti-mouse CD28 (Clone 37.51, BioLegend #102102) antibodies, at the final concentration of 2 μg/ml in PBS. The plates were incubated at 37 °C for at least 1h. Murine CD8^+^ T cells were isolated from spleens of healthy animals, using the EasySep Mouse CD8+ T Cell Isolation Kit (Stemcell #19853), centrifuged 300*g* for 7 min at RT, and stained with a CFSE solution, prepared accordingly to the manufacturer’s protocol (CellTrace CFSE Cell Proliferation Kit, Invitrogen #C34554). The cells were incubated at 37 °C for 3 min in dark, and then the staining was stopped by filling up the tubes with warm medium. The cells were spun down at 300×*g* for 7 min at RT and resuspended in complete medium, then counted and taken up at 625,000 cells/ml. In the meantime, NUP-MDSC were centrifuged 300×*g* for 7 min at RT, the pellet was resuspended in medium, and then the cells were counted and taken up at the final concentration of 625,000 cells/ml. After the antibody coating of the plate, T cells were seeded either alone or in the presence of NUP-MDSC (20 μl/well for each cell type) and incubated at 37 °C, 5% CO_2_ for 72h. After the incubation, samples were acquired using iQue Screener Plus, to assess cell proliferation by measuring CFSE signal. Data analysis was performed using iQue Forecyt software (Sartorius AG, https://www.sartorius.com/en/products/flow-cytometry/flow-cytometry-software).

### Primary Mouse MDSC Screen

Compound source plates (REMP-PP#180030) were pre-prepared by adding 500 nl compound solution (5 mmol/l starting concentration) and 11.5 μl Hepes (20 mmol/l) buffer to achieve a final ready to screen concentration of 0.2 mmol/l. Cocultured cell assay plates were prepared by dispensing 20 μl of differentiated MDSC cells (12,500 cells/cavity) and 20 μl of CD8+ T-cells with a MultidropCombi (12,500 cells/cavity). In the next step, 2 μl of pre-prepared compound solution was transferred *via* BC i5 to the cell plate followed by 3days incubation at 37 °C and 5% CO_2_. Afterward, the supernatant in cell plates were homogenized *via* orbital shaking at 2000 rpm for 20s and centrifugated with 1650 rpm for 30 s. On a BC i5 pipetting robot, 2 μl supernatant were transferred to a pre-filled (30 μl PBS) assay plate followed by centrifugation and addition of 8 μl IFNγ HTRF detection kit, followed by orbital mixing at 2000 rpm for 20s and an incubation step for 3 days at 37 °C and 5% CO_2_. Assay plate was analyzed on an PE Envision. Cell plates were centrifuged in parallel with 300*g* for 60 s and orbital mixed at 2000 rpm for 20 s followed by proliferation analysis *via* Sartorius IQue2. Effects were normalized against stimulator control mIL-2 and 0.2% dimethyl sulfoxide (DMSO) as neutral control. A hit was defined as a compound with >50% effect in at least one of the measured effects.

### Secondary Mouse MDSC Screen

Compound source plates (REMP-PP#180030) for dose-dependent secondary screens were pre-prepared by adding 1000 nl compound solution (1.0E + 01 mmol/l, 3.2E + 00 mmol/l, 1.0E + 00 mmol/l, 3.2E-01 mmol/l, 1.0E-01 mmol/l, 3.2E-02 mmol/l, 1.0E-02 mmol/l, 3.2E-03 mmol/l, 1.0E-03 mmol/l, 3.2E-04 mmol/l; starting concentration) and 12 μl Hepes (20 mM) buffer to achieve a final ready to screen concentration of 4.0E-02 mmol/l, 1.3E-02 mmol/l, 4.0E-03 mmol/l, 1.3E-03 mmol/l, 4.0E-04 mmol/l, 1.3E-04 mmol/l, 4.0E-05 mmol/l, 1.3E-05 mmol/l, 4.0E-06 mmol/l, and 1.3E-06 mmol/l. All other steps are identical to the described workflow under “Primary mouse MDSC screen”.

### Human PMN-MDSC Differentiation Protocol

CD34+ cells were isolated from fresh human blood from healthy donors, obtained from the Health Services unit of the Healthcare Business of Merck KGaA, Darmstadt, Germany. First, peripheral blood mononuclear cells (PBMC) were isolated *via* density centrifugation, and then the target cells were isolated *via* magnetic bead labeling using the EasySep Human CD34 Positive Selection Kit II (Stemcell Technologies #17856). After isolation, the CD34+ cells were cultivated in cultivation medium (StemSpan SFEM II (Stemcell Technologies #09655) + 10% (1×) StemSpan CD34+ Expansion (Stemcell Technologies #02691)) for 10 to 12 days. At that point, the media was exchanged for fresh RPMI 140 medium (Gibco #31870), supplemented with 10% FCS, 2 mM L-glutamine, 100 U/ml penicillin/streptomycin, 1 mM sodium-pyruvate, 1 mM non-essential amino acids, 50 μM ß-mercaptoethanol, and 100 ng/ml human G-CSF (PeproTech, AF-300-23), and the cells were incubated for 10 days in order to derive human PMN-MDSC.

### Human Dendritic Cell Differentiation Protocol

Monocytes were isolated from fresh human blood from healthy donors, obtained from Health Services unit of the Healthcare Business of Merck KGaA, Darmstadt, Germany. First PBMC were isolated *via* density centrifugation, and then the target cells were isolated *via* magnetic bead labeling using the EasySep Human Monocyte Enrichment Kit without CD16 depletion (Stemcell Technologies #19058). After isolation, monocytes were allowed to adhere for 30 to 60 min at 37 °C in RPMI 1640 medium, supplemented with 10% FCS, 2 mM L-glutamine, 100 U/ml penicillin/streptomycin. The cells were then washed and differentiated for 6 days in cultivation medium: RPMI 1640, supplemented with 10% FCS, 2 mM L-glutamine, 100 U/ml penicillin/streptomycin, 20 ng/ml human GM-CSF (PeproTech # AF-300-03), and 20 ng/ml human IL-4 (PeproTech # AF-200-04). To mature the dendritic cell (DC), 0.1 μg/ml of LPS-B5 Ultrapure (InvivoGen # Tlrl-pb5lps) was added 24 h before assay setup.

### Human PMN-MDSC Assay

PMN-MDSC and DC were differentiated as described above. Human CD8+ cells were isolated from the same donor as CD34+ cells, following the isolation of the latter. The CD8+ T cell isolation was performed using EasySep Human T Cell Isolation Kit (Stemcell Technologies # 17951). The cells were then frozen and brought back in culture 1 day before use. On the day of the assay, 60,000 PMN-MDSC were seeded together with 60,000 CD8+ T cells from the same donor and 12,000 DC from a different donor in a 384-well plate (Greiner Bio-One #781090). Where appropriate, 10 μM compounds were added. Control wells, including CD8+ T cells and DC only, were also included. The cells were incubated for 4 days at 37 °C and 5% CO_2_. At that point, the supernatant was transferred and IFNγ was measured using a human INFγ HTRF kit (Cisbio #62HIFNGPEH) following standard assay protocol provided by the manufacturer. The data was analyzed using Genedata software (GeneData Inc, https://www.genedata.com/) with 0% defined by the DMSO-treated wells and 100% effect defined by the wells without PMN-MDSC. Each compound was tested in >20 donor combinations.

### Global Proteome and Phosphoproteome Experiments

#### Cell Differentiation and NUP/NUP-MDSC Characterization

NUP-MDSC were generated as described before and the expression of cell surface markers was checked using flow cytometry. For the characterization of NUP-derived mouse MDSC, five individual differentiation were conducted and cells were harvested and washed twice with PBS before they were subjected to proteomics sample preparation.

#### Compound Treatment of Cells for Proteomic Compound Characterization

For the treatment with the HDAC inhibitors entinostat and mocetinostat, NUP-MDSC were generated as described before and plated in 6-well plates. Next, cells were treated with DMSO, 30 μM, or 300 μM of entinostat or mocetinostat, respectively, for 72 h.

For the treatment with compounds 2 and 3, cells were plated in a 6-well plate and incubated with 3x EC50 (EC50 = 2.6 μM, based on INFγ secretion of secondary mouse screen) of compound 2 or 10 μM compound 3 for 72 h at 37 °C and 5% CO_2_.

After treatments, medium was removed, cells were washed twice with PBS, and the cell pellets were further subjected to proteomic sample preparation.

#### Proteomic Sample Preparation

The cell pellet was reconstituted in 2% SDS in 50 mM Tris–HCl pH 7.5 and lysed by heating to 95 °C for 10 min. One microliter of 100% TFA was added to each sample to hydrolyze DNA, and the pH was subsequently adjusted to 8.5 with 3 M Tris solution. Disulfide bonds were reduced with 10 mM DTT for 45 min at 37 °C, followed by alkylation of cysteines with 55 mM CAA for 30 min at room temperature. Prior to tryptic digestion, the detergent was removed from lysates by SP3 cleanup on an Agilent BRAVO system (Agilent Technologies), following the protocol first described by Hughes *et al*. (34). Briefly, lysate containing 200 μg of protein was mixed with SP3 beads, and proteins were precipitated onto a 50:50 mixture of Sera-Mag Speed Bead types A and B (Thermo Fisher Scientific) in 58% acetonitrile. Beads were washed three times with 80% ethanol in water and once with acetonitrile. Tryptic digest was performed in 100 μl of digestion buffer (2 mM CaCl_2_ in 25 mM Hepes, pH 8.5) with a 1:50 enzyme-to-protein ratio. Bead-precipitated proteins were digested at 37 °C overnight. On the next day, the supernatant of each sample was transferred to a new well and vacuum dried.

Dried peptides were reconstituted in water and labeled with the respective TMT10plex-labeling reagent as described previously (35) with a protein to tandem mass tag (TMT) reagent ratio of 1:2. Labeled samples were pooled, vacuum dried, and reconstituted in 0.1% formic acid (FA) prior to desalting on SepPAC50 columns (waters corp.). Peptides were fractionated using a Dionex Ultra 3000 HPLC system with a Waters XBridge BEH130 C18 3.5 um 2.1 × 250 mm column as described previously (36). Ninety-six fractions were collected and pooled to 48. For the full proteome analysis, one fifth of each fraction was transferred to a new 96-well plate and both plates were vacuum dried. For the phosphopeptide analysis, dried peptides were reconstituted in 55 μl 0.1% TFA in 30% ACN, pooled from 48 to 12 fractions, and mixed with 70 μl priming buffer (0.1% TFA, 100% ACN). Phosphopeptides were enriched from each of the 12 fractions using Fe(III)-IMAC-NTA cartridges on the AssayMAP BRAVO (Agilent Technologies) platform as described previously (36). Phosphopeptides were eluted with 60 μl 1% ammonia, vacuum dried, and stored at −20 °C prior to LC-MS/MS analysis.

#### LC-MS/MS Data Acquisition

The full proteome fractions were measured with a 25 min linear gradient from 4% to 32% acetonitrile at a flow rate of 50 μl/min on a Dionex UltiMate 3000 RSLCnano System coupled online to an Orbitrap Fusion Lumos mass spectrometer (Thermo Fisher Scientific) as described previously (36). The Fusion Lumos was operated in data-dependent acquisition and positive ionization mode. Samples were measured employing a MS3 method. In brief, full scan MS1 spectra were recorded in the orbitrap (OT) from 360 to 1560 m/z at 60,000 resolution using an automatic gain control (AGC) target value of 4e5 charges and a maximum injection time (maxIT) of 50 ms. Iontrap-MS2 spectra for peptide identification were acquired using higher energy collision dissociation (HCD) fragmentation with 32% normalized collision energy (NCE), an isolation window of 0.6 *m/z*, and an AGC target value of 1.2e4 charges. To obtain quantitative information on TMT reporter ions, each peptide precursor was fragmented again as for MS2 analysis followed by synchronous selection of the up to eight most intense peptide fragments in the iontrap (37) and further fragmentation *via* HCD using a NCE of 55%. The MS3 scan was recorded in the OT at 50,000 resolution (scan range 100–1000 *m/z*, isolation window of 1.2 *m/z*, AGC of 1e5 charges, maxIT of 86 ms). The cycle time was 1.2 s, and the dynamic exclusion lasted for 50 s.

The phosphoproteome fractions were measured with 90 min linear gradient from 4% to 32% ACN on a Dionex 3000 (Thermo Fisher Scientific) coupled online to an Orbitrap Fusion Lumos mass spectrometer (Thermo Fisher Scientific). Samples were resuspended in loading buffer containing 0.1% FA. Peptide loading and washing were done on a trap column (100 μm i.d. x 2 cm, packed in-house with Reprosil-Pur C18-GOLD, 5 μm resin, Dr Maisch) at a flow rate of 5 μl/min in 100% loading buffer (0.1% FA) for 10 min. Peptide separation was performed on an analytical column (75 μm i.d. x 40 cm packed in-house with Reprosil-Pur C18, 3 μm resin, Dr Maisch) at a flow rate of 300 nl/min (solvent A: 0.1% FA, 5% DMSO in HPLC grade water; solvent B: 0.1% FA, 5% DMSO in ACN) (38). The Fusion Lumos was operated in DDA and positive ionization mode. Samples were measured employing a MS3 method. In brief, full scan MS1 spectra were recorded in the OT from 360 to 1300 m/z at 60,000 resolution using an AGC target value of 4e5 charges and a maxIT of 50 ms. The top 10 IT-MS2 spectra for peptide identification were acquired in the OT at 15,000 resolution for 22 ms after collisional dissociation by resonance activation with a q-value of 25, using an isolation window of 0.7 *m/z*, and an AGC target value of 5e4 charges. To obtain quantitative information on TMT reporter ions, each peptide precursor was fragmented again as for MS2 analysis followed by synchronous selection of the up to 10 most intense peptide fragments in the IT (37) and further fragmentation *via* HCD using an NCE of 55%. The MS3 scans were recorded in the OT at 50,000 resolution (scan range 100–1000 *m/z*, isolation window of 1.2 *m/z*, AGC of 1.2e5 charges, maxIT of 120 ms). The dynamic exclusion was set to 90 s.

#### LC-MS/MS Data Processing

Peptide, protein, and phosphorylation site identification and quantification were performed with MaxQuant software (v. 1.6.3.3, https://www.maxquant.org/) with standard settings for reporter ion MS3 unless described otherwise (39). Isotope impurities of the TMT lot were specified to allow MaxQuant the automated correction of TMT intensities. Raw files for full and phosphoproteome analysis were searched separately against the mouse reference proteome (UP000000589, canonical, download 10/2019, 55,029 entries). Carbamidomethylated cysteine was set as fixed modification and oxidation of methionine, N-terminal protein acetylation, and phosphorylation of serine, threonine, or tyrosine (only phosphoproteome samples) as variable modifications. Trypsin/P was specified as the proteolytic enzyme with up to two missed cleavage sites allowed. Results were adjusted to 1% false discovery rate (FDR) for peptide spectrum, protein, and site match, employing a target-decoy approach using reversed protein sequences. Furthermore, default settings for the matching tolerances were used, which are 4.5 ppm for precursors, 20 ppm for MS/MS (FTMS), and 0.5 Da for MS/MS (ITMS).

#### Data Analysis

MaxQuant output tables were filtered for contaminants, reversed sequences, and proteins only identified based on modified peptides. For the NUP-MDSC profiling experiment, protein abundance estimation was based on corrected TMT reporter intensities and the TMT intensities for each sample were total sum normalized. Phosphorylation site intensities were normalized with the full proteome total sum intensity ratios. Unless stated otherwise, displayed protein abundances were log_2_ transformed. Statistical analysis was performed with the student’s *t* test function in Perseus (v.1.5.3.3, (40)) using a permutation-based FDR of 0.05 and calculated S0 value (41). Slight modifications were made for the proteomics experiment investigating the effects of compound 2 and 3 as well as the treatment with the HDAC inhibitors. The corrected TMT intensity of each channel was median-centered to the median intensity across all TMT channels. Since the proteome profiling experiment of compounds 2 and 3 were split over three separate TMT plexes, a common bridge channel (MDSC without treatment) was incorporated into each TMT plex and used for normalization to remove batch effects. Statistical analysis was performed with the Student’s *t* test function in Perseus (v.1.5.3.3, ^40^) using a Benjamini-Hochberg–corrected FDR of 0.05 and calculated S0 values. GO term enrichment (GOBP, KEGG, Keywords) was also done in Perseus. The upset plot was created with UpSetR (42).

### Chemoproteomic Competition Assay

#### Synthesis of iCor

Corin (1 μmol) was reacted with DMSO-washed N-hydroxysuccinimide (NHS)-activated (#GE17-0906-01, ∼20 μmol/ml beads) sepharose beads (1 ml) and triethylamine (20 μl) in DMSO (2 ml) on an end-over-end shaker overnight at RT in the dark. Aminoethanol (50 μl) was then added to inactivate the remaining NHS-activated carboxylic acid groups. After 16 h, the beads were washed with 10 ml DMSO and 30 ml EtOH to yield iCor, which was stored until further use at 4 °C in EtOH.

#### Synthesis of iEnt

Entinostat (1 μmol) was reacted with DMSO-washed NHS-activated (#GE17-0906-01, ∼20 μmol/ml beads) sepharose beads (1 ml), triethylamine (20 μl), diisopropylethylamine (10 μl) in DMSO (2 ml) on an end-over-end shaker overnight at RT in the dark. Aminoethanol (50 μl) was then added to inactivate the remaining NHS-activated carboxylic acid groups. After 16 h, the beads were washed with 10 ml DMSO and 30 ml EtOH to yield iCor, stored at 4 °C in EtOH.

#### Competition Pulldown Assays

In vitro–differentiated NUP-MDSC were harvested, washed twice with PBS, and cell pellets were stored at −80 °C. For lysis, the pellets were slowly thawed at 20 °C and mixed with lysis buffer (0.8% Igepal, 50 mM Tris–HCl pH 7.5, 5% glycerol, 1.5 mM MgCl_2_, 150 mM NaCl, 1 mM Na_3_VO_4_, 25 mM NaF, 1 mM DTT, and supplemented with protease inhibitors (SigmaFast, Sigma) and phosphatase inhibitors (prepared in-house according to phosphatase inhibitor cocktail 1, 2, and 3 from Sigma-Aldrich)). Before usage, the cell lysates were cleared from debris by ultracentrifugation (52,000×*g* for 20 min at 4 °C); protein concentration was determined by Bradford assay.

For the target affinity profiling of entinostat, 0.3 ml of NUP-MDSC cell lysate (adjusted to 4 mg/ml protein concentration and 0.4% Igepal) was pre-incubated with eight doses of inhibitor (DMSO vehicle, 10 nM, 30 nM, 100 nM, 300 nM, 1000 nM, 3000 nM, and 30,000 nM) for 1 h at 30 °C in an end-over-end shaker, followed by incubation with 18 μl affinity matrix (iEnt or iCor) (43) for 30 min at 30 °C in an end-over-end shaker. To assess the degree of protein depletion from lysates by the affinity matrix, a second pulldown (PDPD) with fresh beads was performed, using the unbound protein fraction from the vehicle control flow through.

The beads were washed (1x 1 ml of lysis buffer without inhibitors and 0.4% Igepal, 2x 2 ml of lysis buffer without inhibitors and 0.2% Igepal), and captured proteins were denatured with 8 M urea buffer, alkylated with 55 mM chloroacetamide, and digested with trypsin as mentioned previously. Resulting peptides were desalted on a C18 filter plate (Sep-Pak tC18 μElution Plate, Waters), vacuum dried, and stored at −20 °C until LC-MS/MS measurement.

#### LC-MS/MS Measurement of Competition Pulldown Assays

Peptides were analyzed *via* LC-MS/MS on a Dionex Ultimate3000 nano HPLC coupled to an Orbitrap Fusion Lumos mass spectrometer (Thermo Fisher Scientific). Peptides were loaded on a trap column (100 μm x 2 cm, packed in house with Reprosil-Gold C18 ODS-3 5 μm resin, Dr Maisch) and washed with 5 μl/min solvent A (0.1% FA in HPLC grade water) for 10 min. Peptides were then separated on an analytical column (75 μm × 40 cm, packed in house with Reprosil-Gold C18 3 μm resin, Dr Maisch) using a 50 min gradient ranging from 4 to 32% solvent B (0.1% FA, 5% DMSO in acetonitrile) in solvent A (0.1% FA, 5% DMSO in HPLC grade water) at a flow rate of 300 nl/min.

The mass spectrometer was operated in data-dependent mode, automatically switching between MS1 and MS2 spectra. MS1 spectra were acquired over a mass-to-charge (m/z) range of 360 to 1300 m/z at a resolution of 60,000 (at m/z 200) in the OT using a maxIT of 50 ms and an AGC target value of 4e5. Up to 12 peptide precursors were isolated (isolation width of 1.2 Th, maxIT of 75 ms, AGC value of 2e5), fragmented by HCD using 30% NCE, and analyzed in the OT at a resolution of 15,000. The dynamic exclusion duration of fragmented precursor ions was set to 30 s.

#### Competition Pulldown Assay Protein Identification and Quantification

Protein identification and quantification was performed using MaxQuant (v 1.6.1.0) by searching the LC-MS/MS data against all canonical protein sequences as annotated in the Swissprot reference database (v03.12.15, 20,193 entries, downloaded 22.03.2016) using the embedded search engine Andromeda. Carbamidomethylated cysteine was set as fixed modification and oxidation of methionine and N-terminal protein acetylation as variable modifications. Trypsin/P was specified as the proteolytic enzyme and up to two missed cleavage sites were allowed. Precursor tolerance was set to 10 ppm and fragment ion tolerance to 20 ppm. The minimum length of amino acids was set to seven and all data were adjusted to 1% peptide-spectrum matches FDR and 1% protein FDR. Label-free quantification (LFQ) (44) was performed with LFQ min. ratio count of 2 and match between runs was enabled.

#### Competition Pulldown Assay Data Analysis

For the competition assays, relative binding was calculated based on the protein intensity ratio relative to the DMSO control for every single inhibitor concentration. EC50 values were derived from a five-parameter nonlinear regression with a fixed top plateau of 1 and variable slope. The obtained EC50 values were multiplied with a protein-dependent correction factor (cf), resulting in the apparent K_d_ value (K_d_^app^). The correction factor is determined by calculating the ratio of the protein intensity of two consecutive pulldowns of the vehicle control sample (cf^HDAC1^=0.36, cf^HDAC2^=0.39) (45). Targets of the inhibitors were annotated manually. A protein was considered a target or interactor of a target if the resulting binding curve showed a sigmoidal curve shape with a dose-dependent decrease of binding to the beads. Additionally, the number of unique peptides and MSMS counts per condition were considered.

### CellROX-Based Detection of ROS Levels

A594 cells were maintained in Fluorobrite DMEM (Thermo Fisher Scientific) supplemented with GlutaMAX (Thermo Fisher Scientific) and 10% fetal bovine serum (PAN Biotech). Cells were seeded onto a 24-well microplate (Ibidi) and treated with DMSO, 7.8 μM compound 2 (3x EC50), 10 μM compound 3, or 300 μM N-acetyl-cysteine for 48 h. After the treatment, cells were incubated with 200 μM tert-butyl hydroperoxide (Sigma) for 2 h to stimulate ROS production. Then, cells were stained with CellROX deep red (Thermo Fisher Scientific) following the manufacturer’s guidelines. CellROX deep red signal was measured on a Leica DMI 6000 B epifluorescent microscope with a Cy5 filter set. Mean signal intensity per cell was determined from >100 cells per condition from three independent treatments.

### Solvent-Induced Protein Precipitation Profiling

#### Cell Lysate Treatment and Precipitation

NUP-MDSCs were differentiated as described above, washed twice with PBS, resuspended in lysis buffer (PBS, 0.4% IGEPAL CA-630, 1× Halt Protease-Inhibitor Cocktail (Thermo Fisher Scientific)), and subjected to three freeze-thaw cycles. The lysate was cleared by centrifugation for 30 min at 18,000*g* and 4 °C. The soluble fraction was diluted to 4 mg/ml and allowed to warm up to room temperature. Equal volumes of lysate were treated in triplicates with 100 x compound stock solutions (in DMSO) or DMSO for 15 min at room temperature (final concentrations: cmpd 2: 7.8 μM (3xEC50) and 26 μM (10xEC50), cmpd 3: 10 μM and 26 μM). Similar to a previous study (46), each sample was divided into eight aliquots to be denatured using different concentrations (8%, 10%, 12%, 14%, 16%, 18%, 20%, 22%) of acetone:ethanol:acetic acid (50:50:0.1) in water for 20 min at 37 °C with vigorous shaking. Aggregated proteins were separated by centrifugation for 45 min at 4 °C and 3900*g*. Next, equal amounts of the soluble fraction were pooled for subsequent proteomic analysis.

#### Proteomic Sample Preparation

Methanol was added to 100 μg of the pooled supernatants to a final concentration of 70% and further processed according to the R2P1 protocol (47), with tryptic digestion performed in 50 mM Hepes (pH 8.5). Elution fractions were combined and dried by vacuum centrifugation. Dried samples were resuspended in deionized water, and TMT labeling was performed according to Zecha *et al*. (35). All 15 samples (5 treatment conditions in triplicates) were labeled with one TMTpro plex. Next, equal amounts were pooled and desalted by C18 solid-phase extraction cartridges (Sep-Pak Vac 1 cc C18 Cartridges, Waters Corp). Desalted peptides were chromatographically separated *via* basic reversed phase chromatography as previously described (36) into 24 fractions, dried down, and stored at −20 °C until further analysis.

#### LC-MS/MS Measurement

Peptides were analyzed *via* LC-MS/MS on a Vanquish Neo UHPLC coupled to an Orbitrap Eclipse Tribrid mass spectrometer (Thermo Fisher Scientific). Peptides were loaded on a trap column (Acclaim PepMap 100, 75 μM × 2 cm, 3 μM particle size, Thermo Fisher Scientific) and subsequently separated on an analytical column (75 μm × 40 cm, packed in house with Reprosil-Gold C18 3 μm resin, Dr Maisch) using a 110 min gradient ranging from 2 to 32% solvent B (0.1% FA, 5% DMSO in acetonitrile) in solvent A (0.1% FA, 5% DMSO in HPLC grade water) at a flow rate of 300 nl/min. The mass spectrometer was operated in data-dependent mode and positive ionization mode. Samples were measured, employing a MS3 method with real-time search. Full scan MS1 spectra were recorded in the OT over a mass-to-charge (m/z) range of 360 to 1300 m/z at a resolution of 60,000 (at m/z 200) using a maxIT of 50 ms and an AGC target value of 4e5 charges. Iontrap-MS2 spectra for peptide identification were acquired using HCD fragmentation with 35% NCE, an isolation window of 0.7 m/z, an AGC target value of 5e4 charges, and a maxIT of 35 ms. To obtain quantitative information on TMT reporter ions, each peptide precursor was fragmented again as for MS2 analysis followed by synchronous selection of the up to 10 most intense peptide fragments in the iontrap (37) and further fragmentation *via* HCD using an NCE of 55%. For the MS3 scan, real-time search was enabled to only trigger a MS3 scan for peptides that are TMT-labeled and can be identified (searched against a mouse database, same as used for maxquant processing). The MS3 scan was recorded in the OT at 30,000 resolution (scan range 110–500 *m/z*, isolation window of 0.7 *m/z*, AGC of 2e4 charges, maxIT of 120 ms). The cycle time was set to 3 s, and the dynamic exclusion lasted for 60 s.

#### LC-MS/MS Data Processing and Analysis

Raw files were searched with Maxquant (v. 2.0.3.0) against the mouse reference proteome (UP000000589, canonical, download 10/2019, 55,029 entries) with standard settings for reporter ion MS3 unless described otherwise (39). Isotope impurities of the TMT lot were specified to allow MaxQuant the automated correction of TMT intensities. Carbamidomethylation of cysteines was defined as a fixed modification, and oxidation of methionine and N-terminal protein acetylation as variable modifications. Trypsin/P was specified as the proteolytic enzyme with up to two missed cleavage sites allowed. Results were adjusted to 1% FDR for peptide spectrum, protein, and site match, employing a target-decoy approach using reversed protein sequences. Furthermore, default settings for the matching tolerances were used, which are 4.5 ppm for precursors, 20 ppm for MS/MS (FTMS), and 0.5 Da for MS/MS (ITMS).

The Maxquant output ProteinGroups.txt was used for further analysis. All protein entries with less than two unique peptides were removed and the reporter ion intensities were then median-centered across all TMT channels. Statistical analysis was performed with the Student’s *t* test function in Perseus (v.1.5.3.3, (40)) using a Benjamini-Hochberg–corrected FDR of 0.05 and calculated S0 values. For bar plots GraphPad Prism (v.8.3.0) was used.

**Fig. 1:**
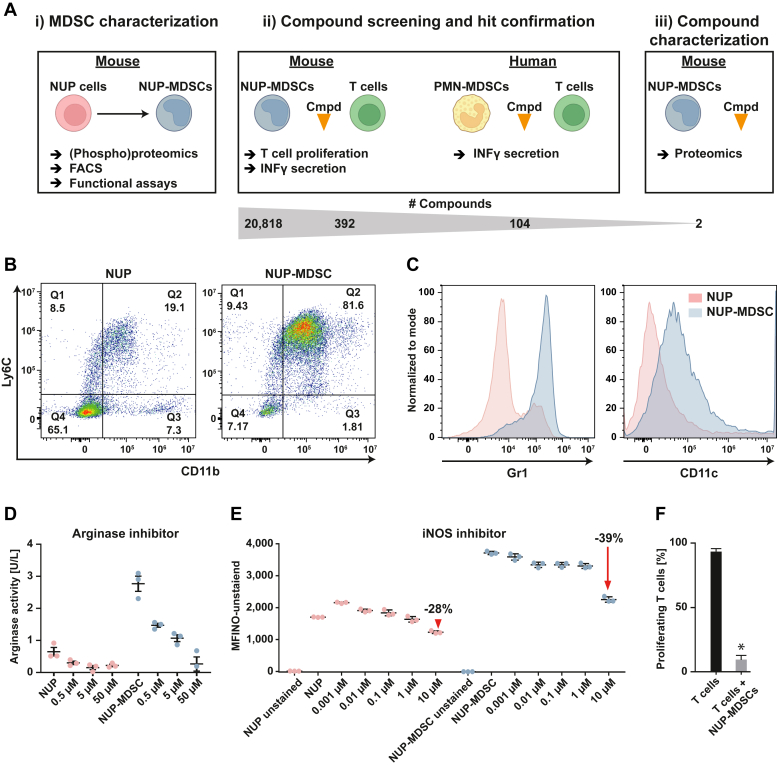
**Differentiated NUP-MDSC possesses phenotypic and functional characteristics of M-MDSC.***A*, schematic overview of experiments performed in this study. *B*, FACS analysis of NUP and NUP-MDSC cells, monitoring the expression of CD11b and Ly6c. *C*, histograms showing expression levels of Gr1 and CD11c in NUP cells and NUP-MDSC. *D*, NUP-MDSC feature increased arginase activity, which can be dose dependently decreased with an arginase inhibitor. *E*, MDSC showed elevated NO production compared to NUPs, and treatment with an iNOS inhibitor can suppress this NO production. *F*, coculturing of T cells with NUP-MDSC suppressed proliferation of the T cell. Asterisk denotes significance (*p*-value <0.0001). iNOS, inducible nitric oxide synthase; MDSC, myeloid-derived suppressor cell; M-MDSC, monocytic MDSC.

**Fig. 2:**
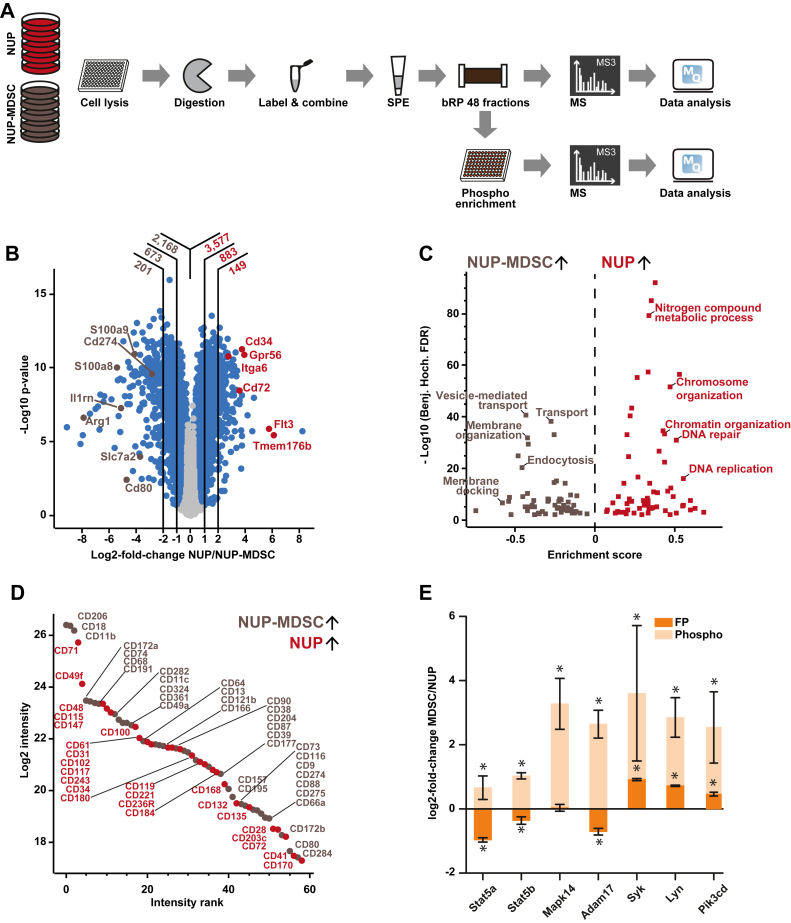
**(Phospho)proteomic characterization of NUP-derived MDSC.***A*, schematic of the (phospho)proteomic workflow. *B*, volcano plot showing differentially expressed proteins between NUP and NUP-MDSC cells. Significantly different proteins are marked in *blue* (*t* test, FDR <0.05). The vertical lines indicate log2-fold changes of 1 and 2, and the numbers at the top indicate how many proteins fall into the respective range. *C*, GO-term enrichment analysis of differentially expressed proteins. *D*, ranked intensity of significantly regulated CD cell surface proteins. *E*, examples of significantly altered phosphorylation sites associated with MDSC. Bars show the average, error bars depict the SD, and asterisks denote significance (*t* test, FDR <0.01). MDSC, myeloid-derived suppressor cell; FDR, false discovery rate.

**Fig. 3:**
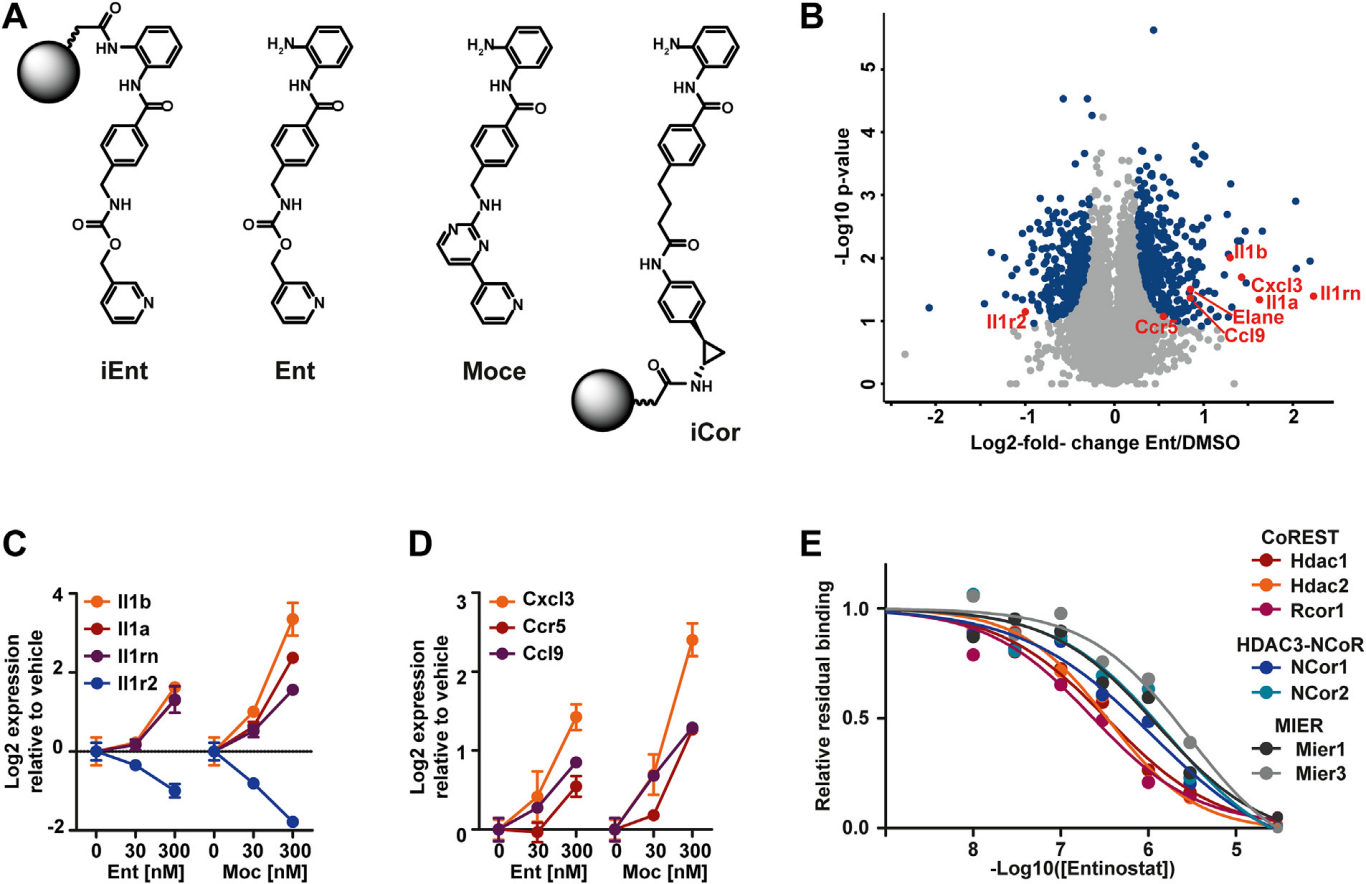
**Effects of entinostat and mocetinostat on *in vitro*–generated MDSC**. *A*, chemical structures of entinostat, mocetinostat, and affinity matrices based on the entinostat pharmacophore. iEnt cannot bind HDACs because the zinc binding warhead is masked. In contrast, iCor retains the zinc-binding group and will pull down HDACs. *B*, volcano plot showing differences in protein expression between entinostat (300 nM) and DMSO-treated cells. *C*, dose-dependent regulation of interleukins and interleukin decoy receptor indicate upregulation of interleukin signaling. *D*, chemokines and chemokine receptors that are dose dependently regulated by HDAC inhibitor treatment. *E*, dose-dependent binding of entinostat to HDACs competes with iCor-mediated pulldown of HDACs and HDAC complexes. HDAC, histone deacetylase; MDSC, myeloid-derived suppressor cell.

**Fig. 4:**
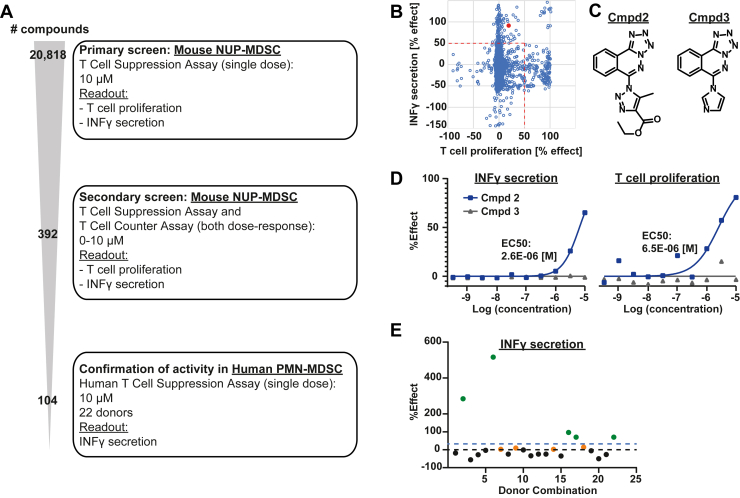
**High-throughput compound screen.***A*, schematic of the screening cascade. *B*, scatterplot showing a summary of the hits identified in the primary screen shown in panel *A*, with % effect on T cell proliferation plotted against % effect on IFNγ secretion. The *red* dot depicts the data for compound 2. *C*, chemical structures of compound 2 and its inactive close analog, compound 3. *D*, dose-response curves for compounds 2 and 3 on IFNγ secretion (*left panel*) and T cell proliferation (*right panel*) with the corresponding EC50 values of compound 2 indicated. *E*, scatterplot showing the % effect of compound 2 on IFNγ secretion in the human PMN-MDSC assay across 23 donor combinations. Donors with % effect> 40% are highlighted in *green*, those with 0 < %effect< 40% in *orange*, and no effect in *black*. MDSC, myeloid-derived suppressor cell.

**Fig. 5:**
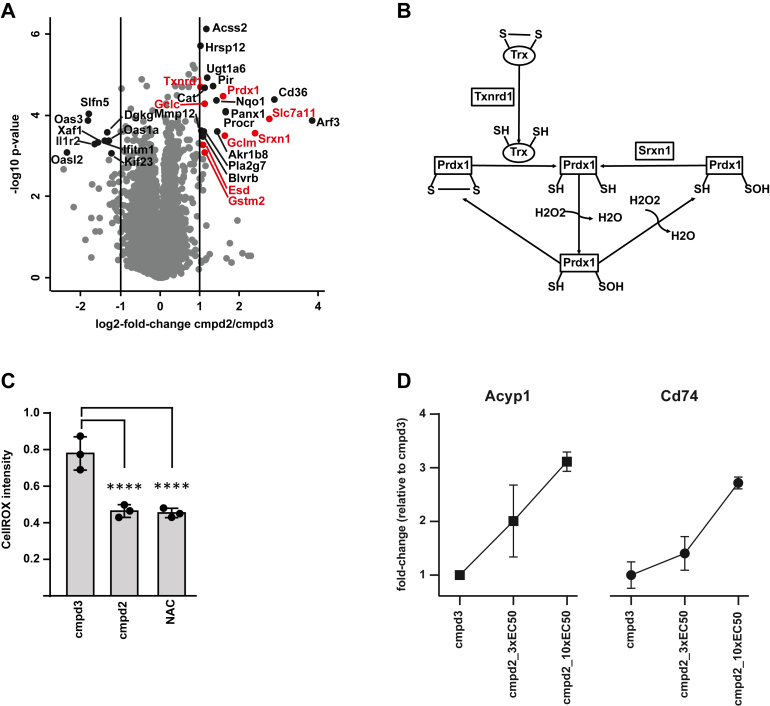
**Compound 2 increases ROS scavenging capacity of MDSC.***A*, differentially expressed proteins upon treatment with compound 2 relative to compound 3. *B*, differentially expressed proteins associated with detoxification by peroxiredoxin. *C*, averaged CellROX intensity of more than 100 cells per replicate (n = 3). Significance was confirmed using one way ANOVA and a post hoc Tukey’s multiple comparisons test (∗∗∗∗*p* < 0.0001). *D*, dose-dependent stabilization against solvent-induced aggregation of Acyp1 and Cd74 upon treatment with increasing doses of compound 2 (3xEC50 = 7.8 μM; 10xEC50 = 26 μM; relative to the inactive compound 3 at 10 μM). Points represent the median for each concentration of compound 2 to the median of the inactive compound 3 and error bars depict the range. MDSC, myeloid-derived suppressor cell; ROS, reactive oxygen species.

### Experimental Design and Statistical Rationale

The T cell proliferation assay was performed as described in the method section. For the statistical analysis, four cells were counted using the iQue Screener Plus and an unpaired *t* test was used to calculate significance.

For the proteome profiling experiment, five independent NUP-MDSC differentiations were performed and harvested after the expression of the cell surface marker CD11b and Ly6C was validated. The LC-MS/MS data was processed with MaxQuant as described in the data processing section. The resulting reporter ion intensities were normalized to the total sum, and entries with valid values for at least three replicates were considered for statistical testing, which was conducted with the Student's *t* test function in Perseus (v.1.5.3.3, S0 calculation based on Tusher *et al*. (41)). The phosphorylation sites were normalized with the sum intensity ratios of the full proteome data and then used for the same statistical analyses. Proteins and phosphorylation sites with a permutation-based FDR below 0.05 were considered significant.

For the proteome expression profiling experiments of NUP-MDSCs treated with HDAC inhibitors, two independent differentiations were performed and validated by the expression of CD11b and Ly6C. Cells from each differentiation were treated with DMSO and two different concentrations of HDAC inhibitors resulting in one TMT10plex experiment. Resulting reporter ion intensities were median-centered to the median intensity across all TMT channels, and each treatment condition was compared to the DMSO controls. To assess significant changes, the Student's *t* test function in Perseus (v.1.5.3.3) was used. The resulting *p*-values were corrected according to Benjamini–Hochberg and classified as significant with an FDR below 0.05. For the global protein expression analysis of compound 2 and 3 treated cells and the solvent-induced proteome profiling experiment, the normalization and calculation of significance was performed in the same way as for the previous profiling experiment. For the global proteome analysis, three independent NUP-MDSC batches were differentiated and treated, while for the solvent-induced proteome profiling, one batch of NUP-MDSC was generated and treated in three independent replicates.

For the CellROX assay, three independent treatments were conducted. From each, the mean signal intensity per cell was determined for >100 cells per condition. The statistical significance was calculated by one-way ANOVA followed by a post hoc Tukey’s multiple comparisons test using GraphPad Prism (v.8.3.0).

For the solvent-induced protein precipitation experiment, one batch of NUP-MDSCs was differentiated and split into three in order to have triplicates for each treatment condition. All 15 samples were labeled with the same TMT plex and the resulting reporter intensities were used for quantification. For normalization purposes, the reporter intensities were median-centered to the median of all 15 channels. Statistical analysis was done with the Student's *t* test function in Perseus (v.1.5.3.3), and the resulting *p*-values were corrected according to Benjamini–Hochberg (FDR < 0.05).

## Results and Discussion

### An *In Vitro* Mouse Model Recapitulates Essential Characteristics of MDSC Biology

We differentiated mouse MDSC from immortalized bone marrow progenitors and used this cellular system to screen ∼21,000 small molecules. The cross-species activity of hit compounds derived from this first screen was further evaluated in a human PMN-MDSC setup. Two molecules were subsequently characterized in more detail by quantitative mass spectrometry–based proteomics to assess protein expression changes upon treatment. A graphical overview of the approach is shown in Figure 1*A*.

In detail, immortalized bone marrow–derived mouse progenitor cells (NUPs, NUP-98-Hoxb4 transgene) were treated with IL-6 and GM-CSF to generate functional NUP-MDSC as described previously (33). After 4 days, the vast majority of cells expressed known markers of the myeloid lineage, such as Ly6C, CD11b, GR1, and CD11c (Fig. 1, *B* and *C*). One well-known mode of action for T cell suppression is the depletion of arginine by arginase 1 and inducible nitric oxide synthase (48). Compared to NUPs, NUP-MDSC showed elevated arginase activity (Fig. 1*D*) and NO production (Fig. 1*E*). Of note, either effect could be decreased using specific arginase or iNOS inhibitors in a dose-dependent fashion. In addition, coculturing of NUP-MDSC and T cells revealed substantially decreased proliferation of the latter (Fig. 1*F*). These data show that the *in vitro* model system mimic general features and biological functions of MDSC and recapitulates essential characteristics of MDSC-mediated T cell suppression (22, 49). Therefore, this system is a promising alternative to primary cells, which are scarce and demanding to culture.

### (Phospho)proteomic Characterization of Mouse MDSC

After validating the functional characteristics of MDSCs, we investigated the molecular characteristics of the *in vitro* MDSC differentiation in more detail. More specifically, we performed a (phospho)proteomic experiment, which resulted in a comprehensive list of differentially expressed proteins, potential MDSC markers, and significantly altered phosphorylation sites (Fig. 2*A* and supplemental Table S1). This data unraveled some of the underlying biology of MDSC generation and can serve as a starting point for novel drug discovery approaches. Briefly, five replicates of NUP cells and NUP-MDSC were harvested, proteins extracted and digested into tryptic peptides, and peptides were stable isotope labeled using TMT. More than 7000 proteins were identified in each replicate. Replicate results were highly correlated and the data contains few missing protein expression values (supplemental Fig. S1*A*), indicating high reproducibility of both, the differentiation protocol as well as the proteomic workflow. Analysis of differentially expressed proteins (*t* test, FDR <0.05) revealed substantial alterations at the proteome level (Fig. 2*B*). Among the differentially expressed proteins, several known MDSC markers, including Arg1, S100a9, S100a8, and Cd274, were significantly upregulated (24, 50, 51). Interestingly, the interleukin 1 receptor antagonist Il1rn, which is a mediator of anti-inflammatory responses and potentially supports the observed suppression of T cells, was more than 30 times upregulated in NUP-MDSC. Moreover, Il1rn is known for inhibiting the activity of interleukin 1, a cytokine which is crucial for proper DC maturation and for T cell expansion (52). A gene ontology analysis revealed a significant increase of proteins related to excretion or vesicle-mediated transport in NUP-MDSC (Fig. 2*C*). As MDSC-mediated T cell suppression is closely associated with vesicles transport and exosomes (reviewed in (53, 54)), this is additional evidence for a successful differentiation and another important feature that can be recapitulated by the *in vitro* model. In contrast, several regulators of immune cell differentiation, such as Tmem176b or Cd72, which drive DC maturation or B cell differentiation, respectively, were substantially decreased in NUP-MDSC (Fig. 2*B*). Similarly, Grp56, a protein controlling HSC maintenance, and Flt3, a kinase associated with the regulation of differentiation and proliferation of hematopoietic progenitors, were significantly less abundant in NUP-MDSC. This apparent decrease of proteins controlling immune cell progenitors is an indication of cell differentiation and thus, the data present a resource of potential protein regulators required for the generation of MDSC. Moreover, the proteomic analysis indicated significantly lower DNA replication activity in NUP-MDSC than NUP cells (Figs. 2*C* and supplemental Fig. S1*B*).

Even though it is not possible to infer cell surface expression of cell surface proteins identified lysate-based experiments directly, the proteome expression data enabled us to determine new putative CD-type marker proteins for NUP and NUP-MDSCs. Apart from the previously known and highly expressed marker proteins Cd11b and Cd206, a list of several differentially expressed CD proteins was discovered (Fig. 2*D* and supplemental Table S1), which can be used in the future as FACS markers to control differentiations or to sort for specific cell populations. Additionally, this list enabled exploring some of the underlying MDSC biology. For example, Cd39 and Cd73, which are strongly linked to immunosuppression (23), were significantly lower expressed in NUP-MDSC and might support the inactivation of T cells.

Since phosphorylation regulates the activity of many proteins and signaling pathways, we analyzed phosphorylation changes during MDSC differentiation. Phosphorylated peptides were enriched by immobilized metal affinity chromatography resulting in the identification of 7437 phosphorylation sites (P-sites) of which 2569 were significantly regulated between the cell populations (fold-change greater than 2; supplemental Fig. S1*C*). In accordance to previous studies reporting high Stat5 signaling in MDSC (55, 56), we observed elevated phosphorylation of the activity-inducing p-site S779 on Stat5a, even though protein levels were lower (Fig. 2*E*). The same trend was observed for S127/128 on Stat5b, which has so far, no reported biological function. Of note, phosphorylation of Mapk14-Y182/T180 indicated a substantial increase in its activity, which was further supported by elevated phosphorylation of Adam17, a downstream substrate of Mapk14. Further kinases with important associations to immune responses and B cell receptor signaling, such as Syk-Y317, Lyn-Y397, and Pik3cd-S1035, were significantly elevated. The list of significantly altered P-sites reported here (supplemental Table S1) is another molecular resource for further mining by the research community.

### Pharmacological Characterization of *In Vitro*–Generated MDSC

Next, we asked if the *in vitro* NUP-MDSC cell system can recapitulate drug effects observed in primary MDSC. To evaluate this, we chose the structurally related HDAC inhibitors entinostat and mocetinostat (Fig. 3*A*), the former of which has shown promising effects when targeting MDSCs (57). Treatment of NUP-MDSC for 72 h with either molecule substantially remodeled the proteomic landscape (Fig. 3*B* and supplemental Fig. S2*A*). At 300 nM, 664 and 4305 proteins were significantly regulated (*t* test, FDR < 0.05) by entinostat or mocetinostat, respectively (supplemental Table S2). Gene set enrichment analysis of the protein abundance changes revealed that both drugs affected histone acetylation–related genes, induced cytostatic effects (indicated by the downregulation of G2M checkpoint proteins), and also significantly altered the expression of immune factors (supplemental Fig. S2, *B*–*H*). Among them were various secreted factors and receptors related to the immune system. These included the interleukin family cytokines Il1a, Il1b, and Il1rn, which were dose-dependently upregulated (Fig. 3*C*) likely as a consequence of the observed downregulation of the decoy receptor Il1r2, which scavenges Il1a, Il1b, and Ilrn (Fig. 3*C*). Moreover, the previously described altered transcription of Cxcr3, Ccl9, and Ccr5 from murine MDSC treated with epigenetic compounds could be confirmed at protein level (58), as they were increased by entinostat and mocetinostat in a dose-dependent manner (Fig. 3*D*).

To be able to attribute the above molecular changes specifically to HDAC inhibition instead of some off-target, we performed chemoproteomic target deconvolution and selectivity profiling for entinostat in NUP-MDSC lysate. Two new affinity matrices were synthesized (iCor: able to bind HDACs; iEnt: unable to bind HDACs; Fig. 3*A*) that better represent the structure of entinostat than previous such chemical tools (43, 59) and were used for competition pull down experiments (supplemental Table S2). Pre-incubation of lysate with free entinostat at different concentrations prior to the pulldown led to a dose-dependent decrease in target protein signal and allowed deriving target affinity values. These experiments confirmed high affinities of entinostat to HDAC1/2 and the CoREST complex and lower affinity to HDAC3, the HDAC3–NCoR complex, or MIER complexes (Fig. 3*E*) in the MDCS model akin to other cell systems. No additional targets of entinostat were identified suggesting that drug-mediated effects in cells can indeed be attributed to HDAC inhibition. Overall, the data show that the cellular system is suitable for drug screening, both from a biological as well as pharmacological perspective.

### High-Throughput Screen for the Identification of MDSC Modulators

After confirming the physiological and pharmacological suitability of the NUP-MDSC model, we developed a high-throughput screen for the identification of potential MDSC modulators (Fig. 4*A* and supplemental Fig. S3). To accomplish this, mouse NUP-MDSC were generated as described before, co-cultured with freshly isolated mouse CD8+ T cells, which were activated with anti-CD3/anti-CD28 antibodies, and subsequently treated with compounds or DMSO vehicle (supplemental Fig. S4*A*). After 72 h, T cell activity was assessed by recording T cell proliferation and IFN-γ secretion. Then, a library of ∼21,000 compounds was screened at 10 μM (Fig. 4*A*, upper panel). Compounds were declared hits if reversing the suppression effect of NUP-MDSC on T cells with ≥50% in either readout resulting in 392 hit compounds (Fig. 4*B*). Hits were subjected to a confirmation screen, where they were tested in a dose-dependent manner using the same assay (Fig. 4*A*, middle panel). In addition, the compounds were tested in a T cell counter assay, where T cell activity (proliferation and IFNγ secretion) was measured in the absence of NUP-MDSC to ensure that the compounds had no direct effect on T cells (supplemental Fig. S4*B*). A compound was considered a validated hit if it showed confirmed activity in the NUP-MDSC assay but had no direct effect in the counter screen, as exemplified by compound 1 (supplemental Fig. S4, *B* and *C*). This led to 104 confirmed hits which were further tested for activity on human MDSC (Fig. 4*A*, lower panel). To assess the ability of these molecules to modulate human MDSC, a PMN-MDSC assay was established (supplemental Fig. S4*D*). Briefly, CD34+ cells were isolated from fresh primary PBMCs from healthy donors and differentiated *in vitro* in the presence of hG-CSF for 10 days. The expression of different PMN-MDSC markers (supplemental Fig. S4, *E* and *F*) and the suppressed cytokine secretion of T cells (supplemental Fig. S4*G*) indicated successful MDSC differentiation. For screening, the PMN-MDSC were cocultured with primary human CD8+ T cells and human DCs for 96h in the presence of DMSO or compound. This setup was used to further evaluate the activity of the 104 hits identified in the secondary screen and to select compounds that exhibited cross-species activity as well as a broad activity on both NUP-MDSC and PMN-MDSC. One such compound was compound 2 (Fig. 4*C*, left panel). Unlike, its inactive close analog compound 3 (Fig. 4*C*, right panel), compound 2 rescued the proliferation and INFγ secretion of mouse CD8+ T cells by suppressing the activity of NUP-MDSC (Fig. 4*D*). To demonstrate its activity on human MDSC, compound 2 was tested in the human PMN-MDSC assay (supplemental Fig. S4*D*) in 23 different donor combinations, in order to capture the diversity of the immune response in patients. In some donors, the compound showed no activity (Fig. 4*E*, black dots); in 4 donor combinations, it had a mild effect of <40% (Fig. 4*E*, orange dots); and in 5 combinations, it had a strong effect of >40% (Fig. 4*E*, green dots). Such activity profiles across different donors are common when working with primary cells and might also represent the diverse effects seen in the clinics.

### Reduction of Reactive Oxygen Species as Potential Mode of Action for Compound 2

The observed cross-species activity made compound 2 an attractive chemical starting point whose mode of action we sought to elucidate using a proteomic approach. NUP-MDSC were treated (in triplicate) with compound 2 and its inactive analog compound 3 for 72 h at 3xEC50 or 10 μM, respectively. Akin to the previous proteomic experiment, cells were harvested and labeled with TMT. In total, ∼8000 proteins were identified with a high overlap between replicates, indicating good reproducibility (supplemental Fig. S5*A*). Differential expression analysis revealed that nine proteins were downregulated upon treatment with the majority being related to immune responses, including Il1r2, Ifitm1, and several members of the Oas family (Fig. 5*A* and supplemental Table S3).

At the same time, 22 proteins were significantly upregulated (Fig. 5*A*). Of note, 13 of these are associated with detoxification functions. Among them are several proteins related to glutathione biology, a crucial antioxidant in cells (supplemental Fig. S5*B*). One example is the cystine/glutamate transporter which supports cysteine uptake, a key component for glutathione synthesis. In addition, Gclc and Gclm, proteins involved in the rate-limiting first step of glutathione synthesis as well as glutathione synthetase, which catalyzes the second synthesis step, were significantly elevated (however only with 20% higher protein level). Moreover, Esd and Gstm2, proteins involved in glutathione-mediated detoxification of formaldehyde or the catalyzes of glutathione binding to electrophilic compounds, respectively, were elevated.

Apart from the glutathione pathway, several proteins associated with detoxification by peroxiredoxin were upregulated (Fig. 5*B*). For example, peroxiredoxin 1 (Prdx1), a protein which converts hydrogen peroxide to water by oxidizing a cysteine residue in its active site. In order to regenerate its detoxification activity, peroxiredoxin is reduced by thioredoxin. Interestingly, the responsible enzyme, thioredoxin reductase 1 (Txnrd1), was significantly upregulated in NUP-MDSC. Moreover, sulfiredoxin 1 (Srxn1), a known activator of peroxiredoxin, followed the same protein expression pattern. Further ROS-reducing proteins were also upregulated, including catalase (Cat), Nqo1, or Blvrb, indicating that treatment with compound 2 may decrease ROS levels, counteracting one of the main effectors for MDSC-derived T cell suppression. Additionally, the protein expression analysis indicates that the treatment did not lead to a de-differentiation of MDSC towards NUP cells as shown by a principal component analysis (supplemental Fig. S5*C*). To test the ROS reduction hypothesis, we quantified the amount of ROS in A594 cells in response to compound. To this end, we treated cells with compound 2, compound 3, and N-acetyl-cysteine, a known ROS scavenger (60), and measured the fluorescence signal of CellROX Deep Red. The fluorescence correlates with the amount of ROS in a cell, which decreased significantly upon treatment with the active compound 2 and the positive control N-acetyl-cysteine (supplemental Fig. S5, *C* and *D*), compared to the inactive compound 3. This further indicates that reduction of ROS is a mode of action of compound 2.

Next, we sought to identify potential targets of compound 2. Unfortunately, it was not possible to generate a chemical probe suitable for affinity-pulldown assays. Therefore, we resorted to using a proteome-wide solvent-induced protein precipitation assay (46). This type of experiment measures the (de)stabilization of proteins that results from protein–compound interactions against solvent-induced protein aggregation. We treated NUP-MDSC cell lysate with compound 2 (7.8 μM, 3× EC50 and 26 μM, 10× EC50) and compound 3 (10 μM and 26 μM), which resulted in a significant stabilization of several proteins for compound 2 (supplemental Fig. S5*E* and supplemental Table S4). The two most strongly stabilized proteins, Acyp1 and Cd74, also showed a dose-dependent effect (Fig. 5*D*) qualifying them as potential targets. Acyp1 is an acylphosphatase that is known to be overexpressed in cancer cells and its levels correlate positively with the abundance of immunosuppressive MDSCs (61). Moreover, it promotes the Warburg effect of cancer cells (62), which has previously been linked to increased ROS levels (63). Cd74 is a cell surface receptor for the cytokine macrophage migration inhibitory factor and plays a crucial role in the maintenance and the differentiation of hematopoietic stem cells (64, 65). Inhibiting CD74, or the interaction of CD74 and migration inhibitory factor, has previously been shown to reduce the number of monocytic MDSC in metastatic lungs (66) and to reduce immunosuppression by enhancing the activity of CD8 T cells (67). Hence, both putative targets have been linked to MDSC biology and function before, and our results indicate that their inhibition might be part of the mode of action of compound 2. Although additional validation experiments are required to characterize this compound, it is an interesting starting point for chemical optimization and may be a useful chemical probe for further research in the field of MDSC.

## Conclusions

In this work, we characterized an in vitro cellular model and developed a phenotypic high-throughput platform utilized in the pharmaceutical industry to screen for novel MDSC modulators. Several new promising candidates with activity in human and mouse MDSC were identified, and proteomic characterization of drug-induced expression changes and target deconvolution experiments enabled us to discover the reduction of cellular ROS as a potential mode of action of one particular hit compound. This work illustrates the value of combining high-throughput phenotypic screening with proteomic follow-up, which can be applied to many other areas of phenotypic drug discovery.

## Data Availability

The mass spectrometry proteomics data have been deposited to the ProteomeXchange Consortium *via* the PRIDE (68) partner repository with the dataset identifier PXD043407. Peaklist were generated using MS-Viewer with the following search keys: Ent_Moce_proteome_profiling: vxoyeec9iz; NUP_MDSC_profiling_phospho: uzsdxns9cl; NUP_MDSC_profiling_proteome: cqlhqyppb4; MDSC_cmpd2_3_treatment_phospho: zae11yniba; MDSC_cmpd2_3_treatment_proteome: uhwliu8rqu.

## Supplemental data

This article contains supplemental data.

## Conflict of interest

The authors declare the following competing financial interests: B. K. is founder and shareholder of OmicScouts and MSAID. He has no operational role in either company. H. H and D. S are employees of OmicScouts GmbH, Freising, Germany. J. K is currently also an employee of OmicScouts GmbH, Freising, Germany. E. P., H.-H. B, A. P., L. K., C. S., L. H., and J. C. P are employees of Merck KGaA, Darmstadt, Germany. The other authors declare no competing interests.
