# Supplementary material for: High-Throughput Screening and Proteomic Characterization of Compounds Targeting Myeloid-Derived Suppressor Cells
Source: Mol Cell Proteomics. 2023 Aug 14;22(9):100632. doi: 10.1016/j.mcpro.2023.100632 (PMC10518717; doi:10.1016/j.mcpro.2023.100632)
Supplement: Supporting information [file mmc5.pdf]

Supporting Information

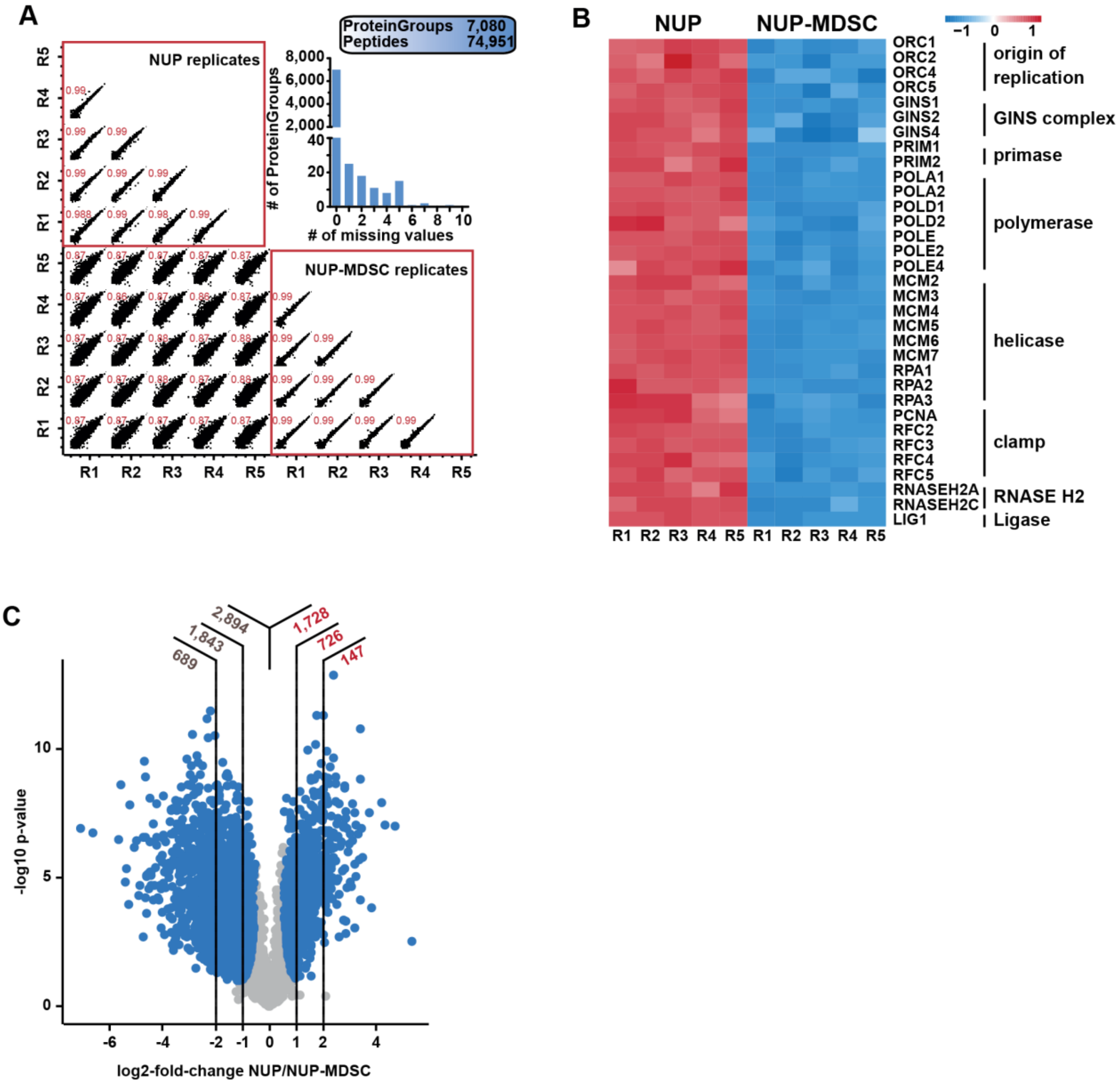

Figure S1 (related to Figure 2): Deep proteomic and phosphoproteomic characterization of mouse MDSC. (A) Scatterplot showing high correlation between replicates and low numbers of missing values (top right panel). (B) Z-scored expression of significantly regulated proteins (t-test, FDR <0.05) associated with DNA replication. (C) Volcano plot of quantified P-sites. Blue colour denotes the significantly altered P-sites (t-test, FDR <0.05). The vertical lines indicate log2-fold-changes of 1 and 2, and the numbers at the top indicate how many proteins fall into the respective range.

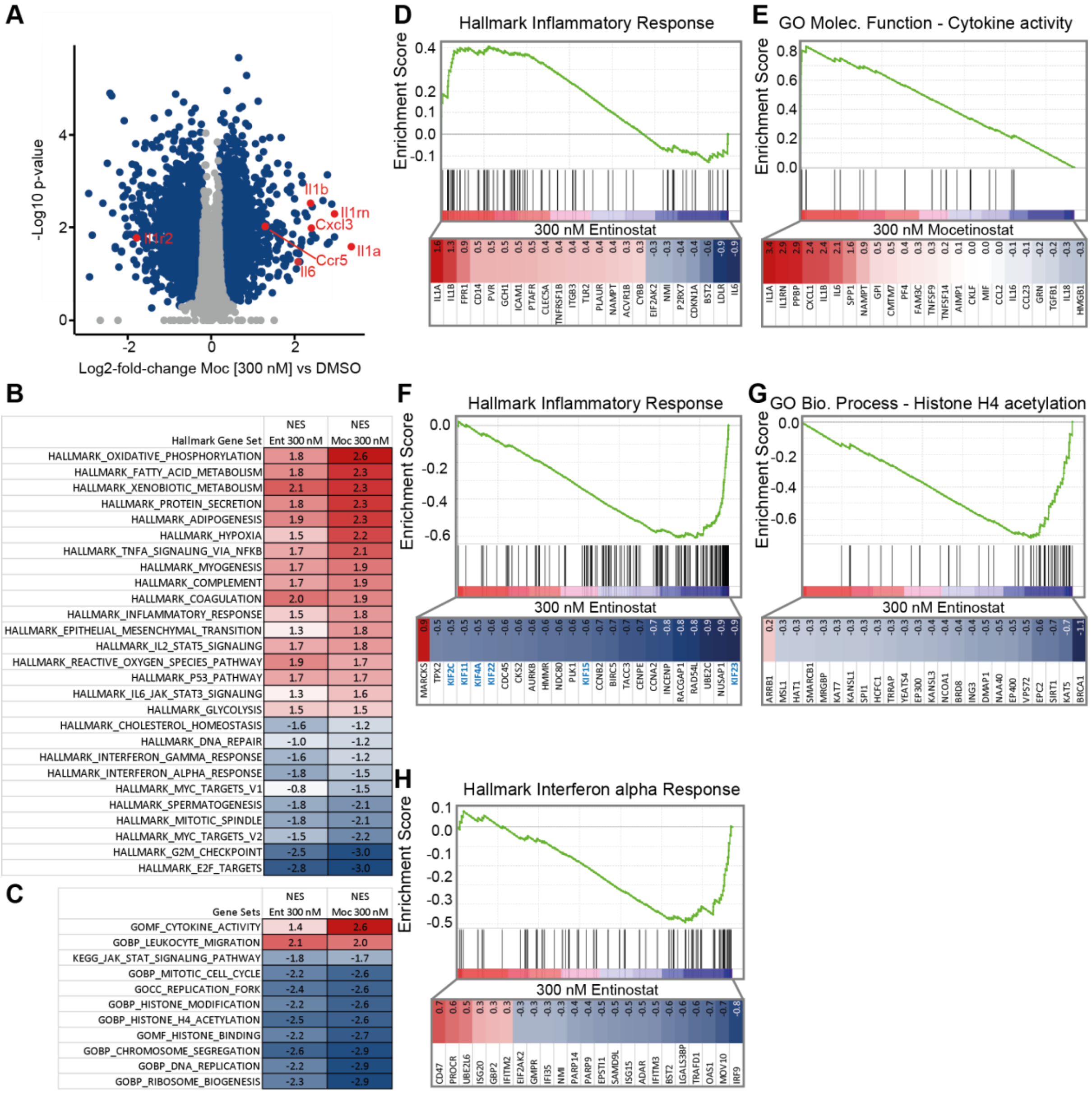

B

Hallmark Gene Set	NES Ent 300 nM	NES Moc 300 nM
HALLMARK_OXIDATIVE_PHOSPHORYLATION	1.8	2.6
HALLMARK_FATTY_ACID_METABOLISM	1.8	2.3
HALLMARK_XENOBIOTIC_METABOLISM	2.1	2.3
HALLMARK_PROTEIN_SECRETION	1.8	2.3
HALLMARK_ADIPOGENESIS	1.9	2.3
HALLMARK_HYPOXIA	1.5	2.2
HALLMARK_TNFA_SIGNALING_VIA_NFKB	1.7	2.1
HALLMARK_MYOGENESIS	1.7	1.9
HALLMARK_COMPLEMENT	1.7	1.9
HALLMARK_COAGULATION	2.0	1.9
HALLMARK_INFLAMMATORY_RESPONSE	1.5	1.8
HALLMARK_EPITHELIAL_MESENCHYMAL_TRANSITION	1.3	1.8
HALLMARK_IL2_STAT5_SIGNALING	1.7	1.8
HALLMARK_REACTIVE_OXYGEN_SPECIES_PATHWAY	1.9	1.7
HALLMARK_P53_PATHWAY	1.7	1.7
HALLMARK_IL6_JAK_STAT3_SIGNALING	1.3	1.6
HALLMARK_GLYCOLYSIS	1.5	1.5
HALLMARK_CHOLESTEROL_HOMEOSTASIS	-1.6	-1.2
HALLMARK_DNA_REPAIR	-1.0	-1.2
HALLMARK_INTERFERON_GAMMA_RESPONSE	-1.6	-1.2
HALLMARK_INTERFERON_ALPHA_RESPONSE	-1.8	-1.5
HALLMARK_MYC_TARGETS_V1	-0.8	-1.5
HALLMARK_SPERMATOGENESIS	-1.8	-2.1
HALLMARK_MITOTIC_SPINDLE	-1.8	-2.1
HALLMARK_MYC_TARGETS_V2	-1.5	-2.2
HALLMARK_G2M_CHECKPOINT	-2.5	-3.0
HALLMARK_E2F_TARGETS	-2.8	-3.0

C

Gene Sets	NES Ent 300 nM	NES Moc 300 nM
GOMF_CYTOKINE_ACTIVITY	1.4	2.6
GOBP_LEUKOCYTE_MIGRATION	2.1	2.0
KEGG_JAK_STAT_SIGNALING_PATHWAY	-1.8	-1.7
GOBP_MITOTIC_CELL_CYCLE	-2.2	-2.6
GOCC_REPLICATION_FORK	-2.4	-2.6
GOBP_HISTONE_MODIFICATION	-2.2	-2.6
GOBP_HISTONE_H4_ACETYLATION	-2.5	-2.6
GOMF_HISTONE_BINDING	-2.2	-2.7
GOBP_CHROMOSOME_SEGREGATION	-2.6	-2.9
GOBP_DNA_REPLICATION	-2.2	-2.9
GOBP_RIBOSOME_BIOGENESIS	-2.3	-2.9

Figure S2 (related to Figure 3): Effects of Entinostat and Mocetinostat treatment on *in vitro*-generated MDSC. (A) Volcano plot showing difference in protein expression between Mocetinostat (300 nM) and DMSO treated cells. (B-H) Gene Set analysis on protein expression changes upon treatment with 300 nM Entinostat or Mocetinostat (NES = Normalized Enrichment Score). (B) Comparison of Entinostat and Mocetinostat effects on expression changes of hallmark gene sets. Shown are gene sets with highest up or downregulation. (C) Comparison of Entinostat and Mocetinostat effects on expression changes of select gene sets from GO and KEGG. (D) 300 nM Entinostat treatment lead to upregulation of inflammatory response associated gene sets (E) epigenetic drug treatment leads to downregulation of genes associated with Cytokine activity. (F) Treatment with Entinostat leads to downregulation of proteins associated to inflammatory responses. (G) HDAC inhibitor treatment downregulates proteins associated with Histone H4 acetylation. (H) Interferon-gamma response related proteins are downregulated by 300 nM Entinostat treatment.

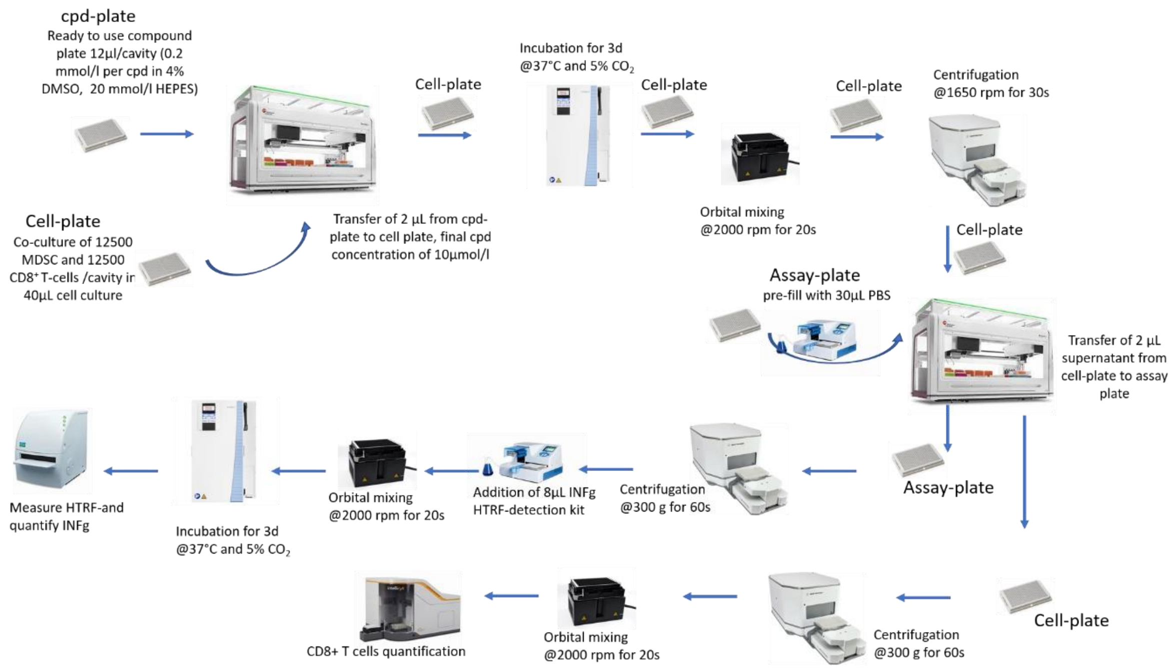

Figure S3 (related to Figure 4): Detailed schematic of mouse NUP-MDSC screening setup.

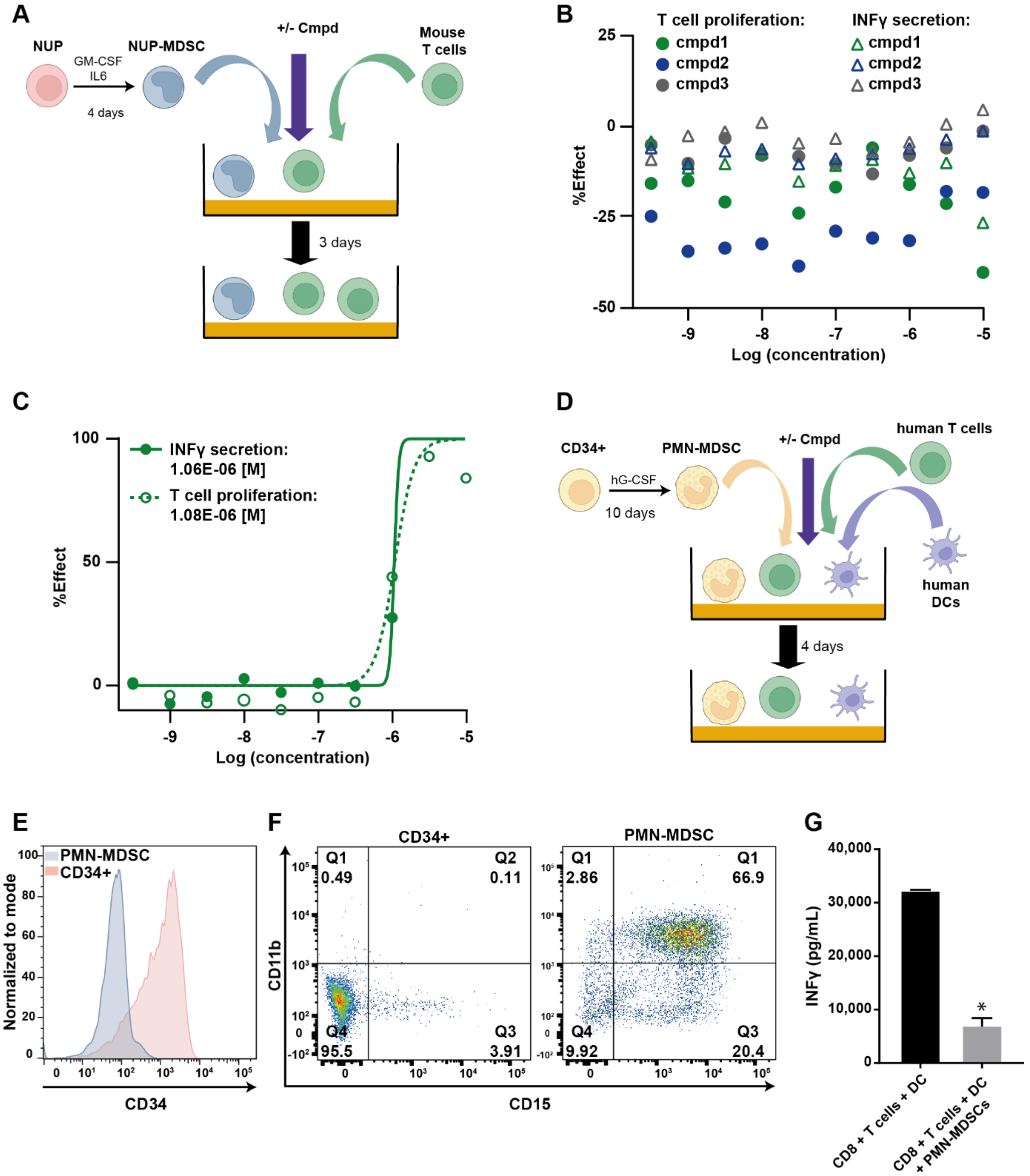

Figure S4 (related to Figure 4): Cell culture system of the mouse and human MDSC screen. (A) Cell culture setup of the mouse MDSC assay. (B) Treatment with compound 1, 2, and 3 in the T cell stimulation assay in the absence of NUP-MDSC. (C) INFγ secretion and T cell proliferation of the control compound 1 for the mouse system. (D) Cell culture setup of the human PMN-MDSC assay. (E) Histograms showing expression levels of CD34 in CD34+ cells (red) and PMN-MDSC (blue). (F) FACS analysis of CD34+ and PMN-MDSC cells monitoring the expression of CD11b and CD15. (G) Reduced secretion of INFγ upon addition of PMN-MDSC. Asterisk denotes significance (p-value <0.0001).

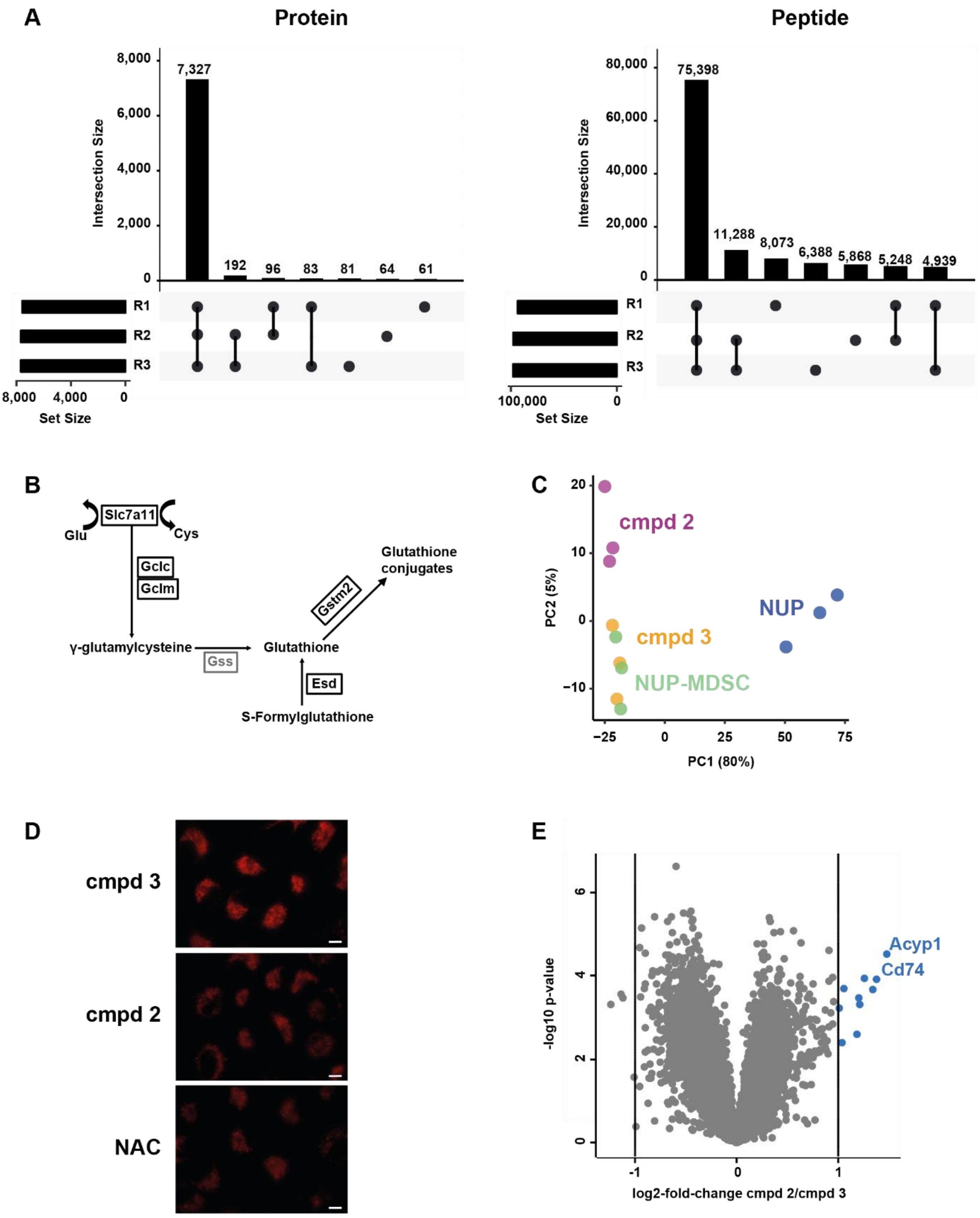

Figure S5 (related to Figure 5): Investigating the mode of action of compound 2. (A) Upset plots showing numbers and overlap of identified proteins and peptides of the proteomic characterization after cmpd 2 or 3 treatment, respectively. R1-R3 relate to the three independently treated replicates. (B) Differentially expressed proteins associated with glutathione synthesis. (C) Principal component analysis of all quantified proteins. (D) Representative fluorescence microscopy pictures of CellROX deep red stained cells after 48h of treatment with cmpd2, cmpd3, or NAC (N-acetyl-cysteine). White scale bar is 10 µM. (E) Volcano plot showing protein stability effects of samples treated with cmpd 2 (10xEC50, 26 µM) compared to cmpd 3 (26 µM). Significant proteins with a log2-fold-change above 1 are depicted in blue.
